# Metastatic myxoid round cell liposarcoma of the buttock: a case report

**DOI:** 10.11604/pamj.2024.48.56.40400

**Published:** 2024-06-13

**Authors:** Niare Ndour, Mamadou Sarr, Assane Diop, Mamadou Diouldé Kanté, Coumba Ndiaye, Astou Diouf, Fatou Diagne, Moussa Diallo, Fatimata Ly

**Affiliations:** 1 Department of Dermatology, Hospital Institute of Social Hygiene, Dakar, Senegal,; 2 Department of Dermatology, Hospital Aristide Le Dantec, Dakar, Senegal,; 3 Laboratory of Dermatopathology of Cheikh Anta Diop University, Dakar, Senegal

**Keywords:** Myxoid liposarcoma, round cell, buttock, metastasis, case report

## Abstract

Liposarcoma is a rare primitive mesenchymal tumor, developed at the expense of adipose tissue and with a preferential location in the thigh. We report an observation of liposarcoma in the buttock. A 56-year-old man, presented with a tumor of the right buttock for 2 years. Examination revealed an inflammatory, ulcerated tumor in the upper-external quadrant of the right buttock, measuring about 8 cm. Bilateral inguinal adenopathies were associated. The diagnostic hypotheses were: a squamous cell carcinoma, a cutaneous lymphoma, and cutaneous metastases. An anatomical examination confirmed the diagnosis of myxoid round-cell liposarcoma. The extension work-up appeared compatible with secondary pleuropulmonary, hepatic, cutaneous, and lymph node neoplastic localizations. The patient was treated with chemotherapy with the Adriamycin-carboplatin protocol. The evolution was rapidly fatal after a few weeks after the first course of chemotherapy. It should be evoked in front of any ulcerated tumor of the buttock.

## Introduction

Liposarcoma is a rare primary mesenchymal tumor developed at the expense of adipose tissue [1]. It consists of 4 histological subtypes, namely well-differentiated, myxoid, pleomorphic, and dedifferentiated [2]. It is preferentially located on the thigh; localization in the buttock is a rare entity [1]. We report a case of a myxoid round-cell liposarcoma in the buttock with metastatic evolution.

## Patient and observation

**Patient information:** a 56-year-old man, a shopkeeper by profession, from Guinea Conakry without any medical or surgical history presented in consultation for a tumor of the right buttock that had been evolving for 2 years. The patient had never been to a hospital and no additional tests had been performed before.

**Figure 1:**
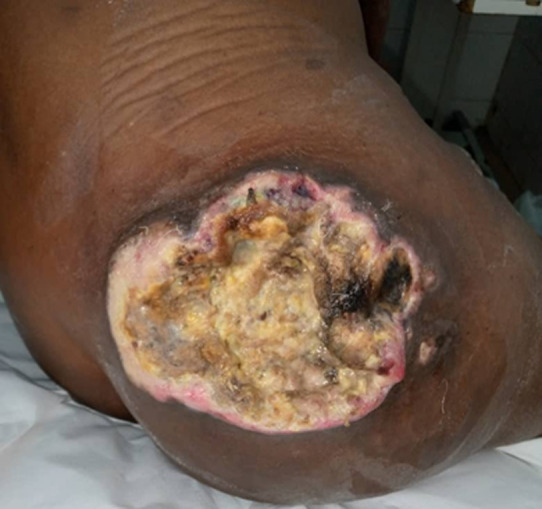
liposarcoma of the buttock. An ulcer of the superior-external quadrant of the buttock, with a purulent background and some necrotic areas

**Figure 2:**
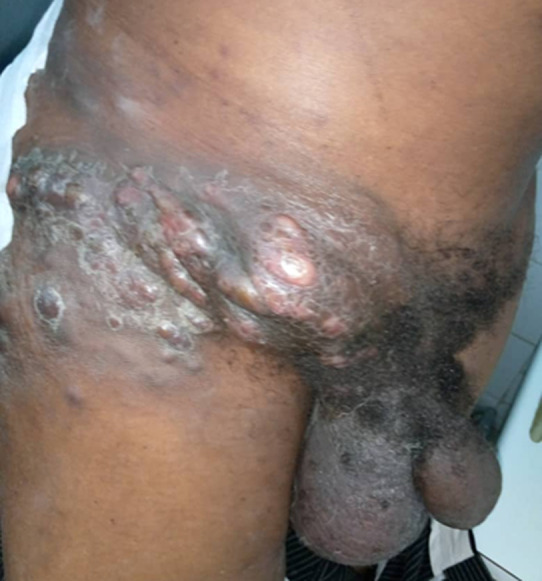
infiltration of the buttock and inguinal region with a large adenopathy

**Clinical findings:** on examination, the patient was in good general condition; he presented an inflammatory, ulcerated tumor of the superolateral quadrant of the right buttock that was well limited, with a yellowish purulent background with some patches of necrosis, measuring about 8 cm (Figure 1). The rest of the examination noted the infiltration of the inguinal region and the presence of bilateral inguinal adenopathies, one of which was large and homolateral to the tumor, measuring 9 cm in long axis (Figure 2).

**Timeline of current episode:** the tumor had been evolving for two years and started as a small nodule that gradually increased in size. When the volume became important, the patient resorted to traditional treatments, and in front of the absence of improvement, he decided to consult in our service.

**Figure 3:**
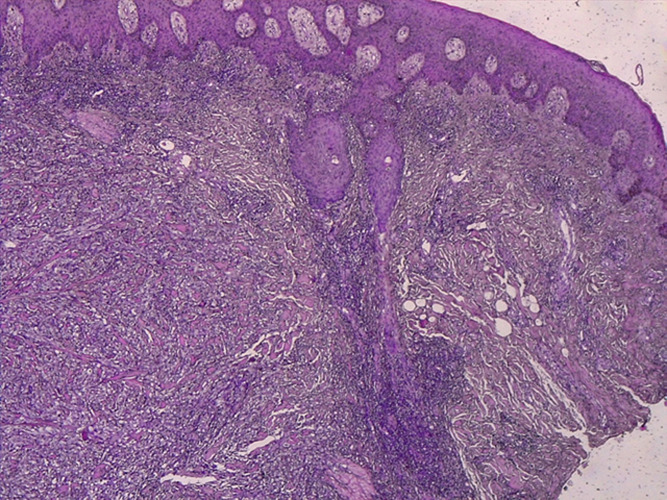
histological image of a myxoid liposarcoma with round cells, pleomorphic

**Diagnostic assessment:** the diagnoses of squamous cell carcinoma and cutaneous lymphoma were evoked and a biopsy of the tumor was performed. The biology revealed a non-specific biological inflammatory syndrome with anemia at 9g/l hypochromic microcytic, an accelerated sedimentation rate at 85 at the first hour and 108 at the second hour, a positive chain reactive protein (CRP) at 151.5 mg/l. Bacteriological sampling of the tumor isolated Pseudomonas aeruginosa sensitive to ceftazidime, aminoglycosides and imipenems. The anatomopathological examination showed a malignant tumor proliferation occupying the whole height of the dermis and made of lipoblasts, bluish mucinous flasks arranged in large areas, and patches of very pleomorphic adipocytes with irregular nuclei with diffuse interstitial arrangement. The diagnosis of myxoid round cell liposarcoma was retained (Figure 3).

**Figure 4:**
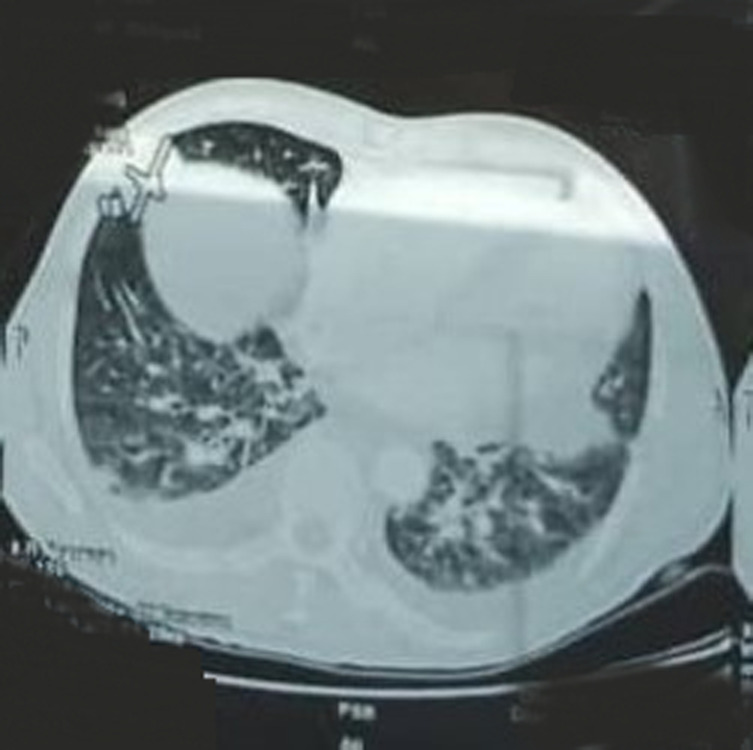
thoracic stage of a CT scan revealed multiple nodules at the upper lobar and bilateral lingular level with a medium lobar macronodule of 39 mm × 18 mm compatible with secondary pulmonary localizations

**Figure 5:**
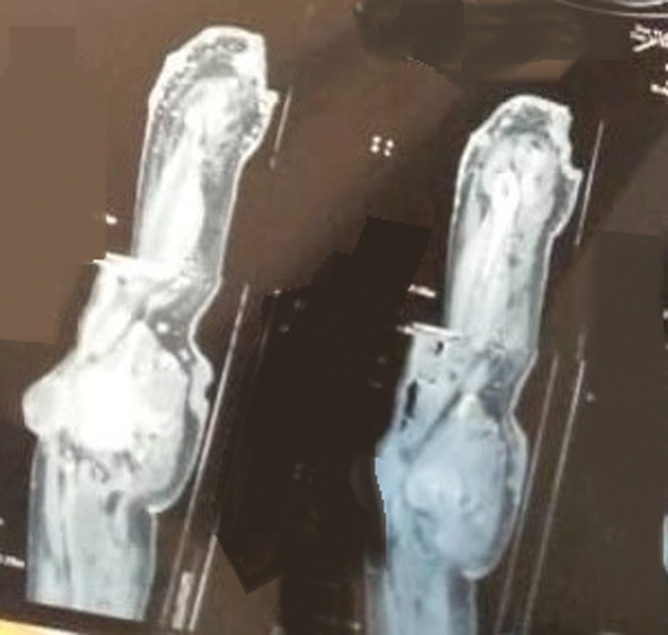
pelvic stage of a CT scan, axial section, showing the buttock infiltrating the soft tissues of the gluteal region, inguinal adenopathies

The thoracic-abdominopelvic CT scan, performed as part of the extension workup, had revealed an appearance compatible with secondary pleuropulmonary, hepatic, cutaneous, and lymph node neoplastic localizations. The pleuropulmonary localizations consisted of multiple nodules at the upper lobar and bilateral lingular level, with a medium lobar macronodule of 39 mm x 18 mm (Figure 4). At the pleural level, there was a small bilateral effusion. The liver involvement consisted of a 20 x 13 mm nodule located in segment 7. The local extension involved the abdominal and pelvic subcutaneous tissues and the muscles of the buttock. The lymph nodes involved the bilateral internal and external inguinal and iliac areas with adenopathies of varying sizes, the right inguinal target measuring 96 x 94 mm (Figure 5). These adenopathies were compressive and complicated by stage II uretrohydronephrosis on the right and partial vena cava thrombosis. In addition, at the abdominal level, the CT scan revealed a small amount of effusion; however, there were no suspicious focal lesions in the pancreas, adrenals, spleen, and kidneys. Bone involvement was not objectified.

**Therapeutic interventions:** the patient was treated by chemotherapy with Adriamycin-carboplatin protocol for palliative purposes, established after multidisciplinary consultation. The dosage of adriamycin was 60 mg/m^2^ for 3 days and carboplatin was administered at a dosage of 400 mg/m^2^, as a short intravenous infusion.

**Follow-up and outcome of interventions:** no side effects were noted in the immediate aftermath of the first course of chemotherapy. However, the patient died in his home a few weeks after the first course of chemotherapy.

**Patient perspective:** during the first course of chemotherapy, the patient was satisfied with the level of care provided to him.

**Informed consent:** written informed consent was obtained from the patient family for participation in our study.

## Discussion

The strong point of our observation is the rare localization of liposarcoma in the buttock and the main limitation was the absence of immunohistochemistry due to a lack of technical support. Also, the delay of therapeutic management had favored the occurrence of metastasis and probably the death of the patient, confirming moreover, the metastatic potential of the round cell myxoid subtype. Liposarcomas are rare malignancies representing less than 1% of all malignancies [1]. In 2002, the World Health Organization recognized four distinct histological types, including well-differentiated liposarcoma (low grade), consisting of mature adipose cells, with a good prognosis because it rarely metastasizes; myxoid liposarcoma (intermediate grade), consisting of primitive non-lipogenic mesenchymal cells, spindle-shaped, in a large homogeneous myxoid stroma with a plexiform vascular network, recurs and quickly metastasizes [2]; round cell liposarcoma composed of homogeneous sheets of round, oval or spindle-shaped cells, it rapidly gives metastases and has a poor prognosis; pleomorphic liposarcoma, rich in cells with very atypical nuclei, nucleated and often in mitosis, with a very poor prognosis because it gives metastases as soon as it is diagnosed [3]; dedifferentiated liposarcoma combines areas of well-differentiated liposarcoma with areas of poorly differentiated liposarcoma.

The myxoid subtype is the most common, accounting for 25% of liposarcomas and 5% of soft tissue sarcomas, usually affecting young adults with a peak incidence between the ages of 40 and 60 years [1,4]. The most recent version of the WHO classification merges liposarcoma myxoid liposarcoma and round cell liposarcoma, since the latter is simply a high-grade variant of the former, and it is common to see a transition between one and the other in the same tumor [1]. Myxoid liposarcoma classically occurs in the deep soft tissues of the extremities, especially the thighs. It is rarely localized to the buttock [5]. Magnetic resonance imaging (MRI) with contrast injection plays a fundamental role in suggesting a diagnosis even before the anatomopathological examination by giving images that may be specific. But this depends on the level of differentiation and the rate of round cells. Indeed, a rate of round cells higher than 5% decreases the specificity of the magnetic resonance imaging (MRI) [6]. It shows a heterogeneous tumor with a fatty component in T1 hypersignal and T2 hypersignal; the myxoid component is suspected in front of a very intense signal in T2 [7]. In our patient, magnetic resonance imaging (MRI) was not performed due to lack of resources. Histologically, it is composed of ovoid cells of relatively small size, the lipoblasts, which may be uni- or multivacuolar, arranged in an abundant myxoid matrix, traversed by a sophisticated capillary network sometimes reminiscent of the meshwork of a fence.

In immunohistochemistry, a marker known to be sensitive and specific has recently been described (NY-ESO-1), but its use is not widespread and its reliability remains to be demonstrated [8]. Genetically, myxoid liposarcoma is associated in 95% of cases with a t (12, 16) translocation that results in a FUS-DDIT3 (formerly FUS-CHOP) juxtaposition. In a small number of cases, a t (12, 22) translocation results in EWSR1-DDIT3 juxtaposition. Several breakpoints exist for each of these translocations, but they do not appear to have significant clinical impact. Fusion transcripts can be detected by RT-PCR and chromosomal rearrangements demonstrated by FISH [9]. Myxoid round cell liposarcoma is a tumor with high metastatic potential and its risk increases with the proportion of “round cell” morphology. Metastases tend to be localized at the skeletal and retroperitoneal level rather than at the pulmonary level with a greater sensitivity of whole-body magnetic resonance imaging in their early detection [6]. In our patient, lesions suspicious of secondary pulmonary localization were already noted at the time of diagnosis, testifying to the severity of this round cell variety and especially aggravated by the diagnostic delay. The treatment of choice for liposarcoma remains excisional surgery with healthy resection margins. Coupled with radiotherapy, this surgery gives a better control rate of 85 to 90% [10]. The role of chemotherapy is controversial and is proposed on a case-by-case basis. Our patient underwent chemotherapy with Adriamycin-carboplatin as a palliative treatment because of the already established metastases. The prognostic factors for recurrence are age, size > 10 cm, R1 margins, round cell count > 5% [6].

## Conclusion

Myxoid round cell liposarcoma of the buttock is a rare entity. Its strong metastatic potential should lead practitioners to evoke it in front of any rapidly evolving tumor of the buttock and to perform a biopsy for anatomopathological and immunohistochemical examination.
